# Addition of Bempedoic Acid to Statin–Ezetimibe versus Statin Titration in Patients with High Cardiovascular Risk: A Single-Centre Prospective Study

**DOI:** 10.3390/jcdd11090286

**Published:** 2024-09-14

**Authors:** Giuseppe Marazzi, Giuseppe Caminiti, Marco Alfonso Perrone, Giuseppe Campolongo, Luca Cacciotti, Domenico Mario Giamundo, Ferdinando Iellamo, Paolo Severino, Maurizio Volterrani, Giuseppe Rosano

**Affiliations:** 1 Cardiology Rehabilitation Unit, Istituto di Ricovero e Cura a Carattere Scinrifico ì San Raffaele, 00163 Rome, Italy; giuseppe.marazzi@sanraffaele.it (G.M.); maurizio.volterrani@sanraffaele.it (M.V.); 2 Department of Human Science and Promotion of Quality of Life, San Raffaele Open University, 00163 Rome, Italy; 3 Division of Cardiology and Sports Medicine, Department of Clinical Sciences and Translational Medicine, University of Rome Tor Vergata, 00133 Rome, Italy; iellamo@uniroma2.it; 4 Department of Cardiac Surgery, S. Carlo di Nancy Hospital, 00165 Rome, Italy; gcampolongo@gvmnet.it; 5 Cardiology Unit, Madre Giuseppina Vannini Hospital, 00177 Rome, Italy; lcuccc@libero.it; 6 Department of Systems Medicine, Tor Vergata University, 00133 Rome, Italy; jamundus20@libero.it; 7 Department of Clinical Internal Anesthesiology and Cardiovascular Sciences, Sapienza University of Rome, 00163 Rome, Italy; paolo.severino@uniroma1.it; 8 Department of Cardiology, St George’s University, St George’s University Hospitals, London SW17 0QT, UK; giuseppe.rosano@gmail.com; 9 Department of Cardiology, San Raffaele Cassino Hospital, 03043 Cassino, Italy

**Keywords:** bempedoic acid, hypercolesterolemia, primary prevention, statins, ezetimibe

## Abstract

Reducing levels of low-density lipoprotein cholesterol (LDL-C) below recommended thresholds is a core component of cardiovascular prevention strategies. We hypothesized that the addition of bempedoic acid to patients already on statin–ezetimibe therapy was more effective than titrating the statin dose in reducing LDL-C. The study enrolled 120 patients at high cardiovascular risk and with LDL-C above 70 mg/dL. They were randomly divided into two groups: the bempedoic acid (BA) group, taking bempedoic acid in addition to statin plus ezitimibe, and the statin titration (ST) group, including patients who doubled the dose of statin. At 12 weeks, the BA group presented a more significant decrease in LDL-C compared to the ST group (−22.9% vs. 7.5% *p* 0.002). The total cholesterol decreased significantly in the BA group compared to ST (−14.8% vs.−4.7%; *p* 0.013) No significant between-group changes in HDL and triglycerides occurred. At 12 weeks, the number of patients who reached LDL-C lower than 70 mg/dL was 38 (63%) in the BA group versus 22 (37%) in the ST group (between groups, *p* 0.034). In the BA group, the LDL-lowering effect of bempedoic acid was similar between patients taking atorvastatin and rosuvastatin. No side effects occurred during the follow up period. In conclusion, the addition of bempedoic acid to statin–ezetimibe combined treatment was more effective than doubling the dose of statin in reducing LDL-C levels and increased the number of patients reaching the LDL-C goal.

## 1. Introduction

Cardiovascular (CV) diseases are the leading cause of death worldwide, being responsible for about 30% of total mortality. Atherosclerotic cardiovascular disease (ASCVD), through its main clinical manifestations that include ischemic heart disease, ischemic stroke and peripheral artery disease, accounts for most CV deaths [1,2]. The current clinical approach to the prevention of ASCVD includes the promotion of a healthy and physically active lifestyle as well as the administration of drugs for treating hypertension, hypercholesterolemia or diabetes whenever indicated [3]. Lowering blood levels of low-density lipoprotein cholesterol (LDL-C) remains a primary target for counteracting the onset and progression of ASCVD. It has been estimated that a 1 mmol/L reduction in LDL-C is associated with a 20–25% reduction in major adverse cardiovascular events and a 10% reduction in all-cause mortality over 5 years [4]. In the context of primary prevention, European guidelines recommend achieving the goal of LDL-C below 70 mg/dL for subjects considered at high CV risk [3]. The first and main option for lowering LDL-C is statins administered at a maximally tolerated dose since they have the most consistent demonstration of reducing CV risk [5,6,7]. American guidelines recommend that clinicians prescribe a statin for the primary prevention of CVD for adults aged 40 to 75 years who have 1 or more CVD risk factors and an estimated 10-year CV risk of 10% or greater [8]. However, there are instances in which non-statin lipid-lowering therapies may be needed. These scenarios include patient unwillingness to take statins, intolerability of statin side effects, particularly when these drugs are taken at high doses, and failure to meet LDL-C goals with statin therapy alone. The association of statin with ezetimibe has been shown to potentiate the LDL-C-lowering effects of statins and partially fill this gap [9,10,11]. However, a significant proportion of patients considered at high risk remain with levels of LDL-C above the recommended threshold despite the combination therapy [12]. There is therefore a need for developing new drugs and pharmacological strategies for these patients. Bempedoic acid is an inhibitor of the hepatic cholesterol biosynthetic pathway by modulating the activity of ATP citrate lyase, resulting in upregulation of LDL receptor expression with improved clearance of LDL and a reduction in blood LDL-C levels [13]. The effectiveness of bempedoic acid in reducing LDL-C levels has already been tested by administering the drug alone, in association with statins or ezetimibe or as a component of a triple therapy, also including statin plus ezetimibe [14,15,16]. The administration of bempedoic acid has shown to be capable of improving strong endpoints by reducing major cardiovascular events in statin-intolerant patients [17]. Despite these encouraging results, the exact role of bempedoic acid in the prevention of ASCVD has not yet been established. In the present study, we explored the potential role of bempedoic acid in patients at high-risk who were already on a comprehensive cholesterol-lowering therapy by taking a high-intensity statin and ezetimibe but who needed further therapeutic implementation not having reached the LDL-C goal. We compared the effects of adding bempedoic acid, for 12 weeks, to their background therapy with the alternative strategy of increasing the dose of statins. The primary endpoint was comparative change in LDL-C from baseline to week 12. The secondary endpoint was comparing the number of patients reaching the target of LDL-C below 70 mg/dL at the end of the study.

## 2. Materials and Methods

### 2.1. Population

The study enrolled 120 patients who were attending the San Raffaele IRCCS cardiology outpatient service from June 2022 to January 2024. The following inclusion criteria were adopted: age over 18 years; stable clinical conditions; previous diagnosis of hypercholesterolemia; being already treated with a combined lipid-lowering therapy including a high-intensity statin plus ezetimibe for at least three months; LDL-C levels persistently (at least in two previous determinations) above 70 mg/dL; no record of previous side effects to statin treatment; no history of cardiovascular and/or cerebrovascular diseases. The following exclusion criteria were adopted: significant renal failure (GFR < 30 mL/min); concomitant chronic liver diseases; recent diagnosis of cancer; patients who had total fasting triglycerides of 500 mg/dL or greater; and patients taking other lipid-lowering drugs such as niacin or nutraceutics products. The study complied with the Declaration of Helsinki and was approved by the local Ethics Committee of San Raffaele IRCCS (protocol number 04/2022). All patients gave written informed consent before entering the study. The Systematic COronary Risk Evaluation (SCORE2) algorithm [18] was used to estimate 10-year risk of fatal and non-fatal (myocardial infarction, stroke) cardiovascular diseases. According to the ESC guidelines, we considered to be at high risk patients between 50 and 69 years with an estimated CV risk of 5 to 10%; patients over 70 years with an estimated CV risk of 7.5 to 15%; and patients under 50 years with an estimated CV risk of 2.5 to <7.5% [3]

### 2.2. Study Design

**Figure 1:**
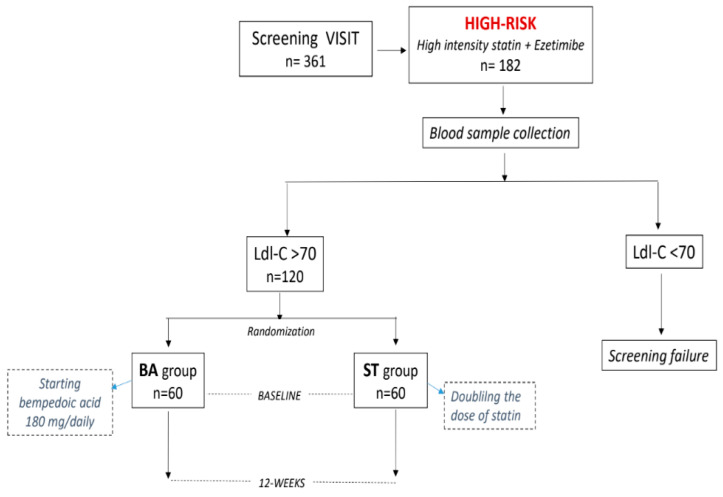
Study flow chart.

The study flow chart has been summarized in Figure 1. The study was conceived as an open randomized trial with two parallel arms. Patients were randomly assigned on a 1:1 basis to one of the following groups: bempedoic acid (BA) or statin titration (ST). Each group was composed of 60 patients. The randomization code was developed by a computer’s random-number generator to select random permuted blocks. The protocol required that patients of the BA group would start bempedoic acid, 180 mg/daily, and would maintain, unchanged, their dose of statin through the study. Patients of the ST group would double the dose of their statin; no other changes in lipid-lowering drugs were allowed during the entire study. At baseline, patients performed a preliminary visit during which inclusion/exclusion criteria were evaluated; medical history and previous blood cholesterol levels and anthropometric parameters were collected. Body mass index (BMI) was calculated through the following formula: BMI = kg/m^2^, where kg is a person’s weight in kilograms and m^2^ is their height in meters squared. During this visit, suitable patients were invited to participate in the study and those who agreed were then summoned for blood sample collection. Blood collections were performed in the morning between 7:30 and 9:30, within a week from the first visit. Patients were required to fast for 12 h before the blood was drawn. The baseline assessment of LDL-C had a confirmatory value: results indicating that patients had LDL-C above 70 mg/dL were considered to be screening failures. For patients entering the study, blood collections were then repeated at 12-weeks at the same time and in the same way as at baseline. Clinical laboratory samples for the analysis of basic fasting lipids (total cholesterol (TC), calculated LDL-C, high-density lipoprotein cholesterol (HDL-C), non-HDL-C and triglycerides). LDL cholesterol concentration was calculated using the Friedewald formula: LDL-c (mg/dL) = TC (mg/dL) − HDL-c (mg/dL) − TG (mg/dL)/5 [19]. Non-HDL cholesterol (non-HDL-C) was defined as the difference between total TC and HDL-C levels. Patients were not asked to change their dietary habits and their lifestyle organization during the study.

### 2.3. Statistical Analysis

Since we did not find in the literature previous studies in which bempedoic acid was administered to patients already taking statin plus ezetimibe, we were unable to establish a proper size effect. Therefore, for the present research, we based the sample size calculation on the data obtained by using bempedoic acid in the setting of primary prevention [17]. We estimated that a sample size of 55 subjects per group had 80% power to detect a difference in LDL-C decrease of 15 percentage points between the two groups, with a standard deviation of 10% using a two-sided significance level of 0.05. We estimated the drop-out rate to be 10%, leading to an overall sample size of 60 patients per group. Data were expressed as median ± standard deviation. The Shapiro–Wilk hypothesis test was used to check the assumption of normality. For each parameter assessed, delta (Δ) was defined as the difference between value at 12 weeks versus value at baseline. Between-group comparisons (Δs BA versus Δs ST) were made by using the *t* test for unpaired groups. Categorical variables were compared by using the chi-square test. The level of significance was set at *p* < 0.05. Data were analyzed by using SPSS software (version 20.0 IBM Corp, Amonk, New York, NY, USA). 

## 3. Results

**Figure 2:**
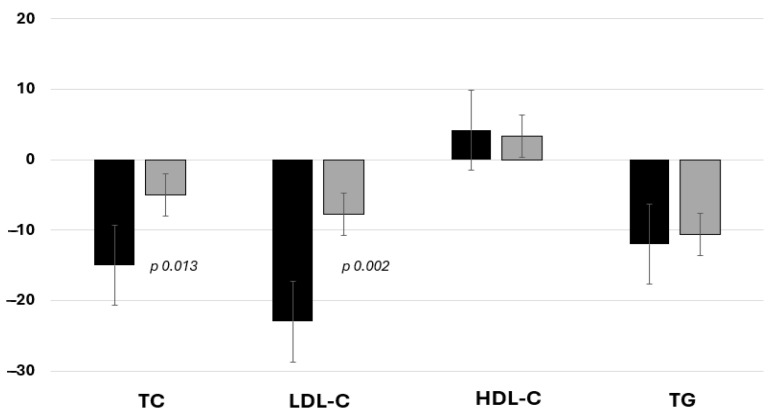
Percentage changes (12 week versus baseline) in lipids in the BA group (dark bars) and ST group (light bars). Serum levels of total cholesterol and LDL cholesterol decreased significantly in the BA group (ac bempedoic + statin + ezetimobe) compared to ST (statin titration + ezetimibe) group after 12 weeks.

From an original population of 361 patients who were initially screened, we selected 182 patients who were at high risk of CV and were taking, at the same time, high-intensity statin and ezetimibe. In total, 120 out of 182 (66%) people were then selected to participate in this study because they had LDL-C levels above the threshold of 70 mg/dL. Out of 120, 67 had at two adjunctive CV risk factors beyond hypercholesterolemia. Overall, 75 were taking at least two anti-hypertensive drugs and 86 out of 120 (72%) were aged between 50 and 69; only 5 patients (4%) were under 50 years of age. More than 50% of the whole sample had diabetes. The baseline features of the population are reported in Table 1. At baseline, the two groups were comparable regarding age, anthropometric data, laboratory parameters, and pharmacological therapy. The average dose of atorvastatin taken by patients was 11.3 mg/daily and that of rosuvatatin 8.2 mg/daily. The baseline LDL-C for the entire population was 88.7 ± 21.7 mg/dL. All patients completed the study. At 12 weeks, the reduction in LDL-C observed in the BA group was significantly greater than in the ST group (between-group change: −13.8 mg/dL, [95%CI= −11.6–15.3] *p* 0.002). The reduction in TC in the BA group was significantly greater than in the ST (between-group change: −9.5 mg/dL, [95% CI = −6.7–12.3], *p* 0.013) (Figure 2). The reduction in non-HDL-C in the BA group was significantly greater than in the ST (between-group change: 16.8 mg/dL, [95% CI = −13.2–19.6], *p* 0.026). Changes in HDL and triglycerides were similar between the two groups (Table 2). At 12 weeks, the number of patients who reached LDL-C values lower than 70 mg/dL was 38 (63%) in the BA group versus 22 (37%) in the ST group (between groups, *p* 0.034). No changes in uric acid, glucose, and creatine kinase occurred in the two groups. Among patients of the BA group, 33 out of 60 (55%) were taking atorvastatin/ezetimibe and the average dose of atorvastatin was 10.6 ± 2.2 mg. The average dose of rosuvastatin was 7.8 ± 4.7. At 12 weeks, in the BA group the reductions in TC and LDL-C were similar between patients taking atorvastatin and rosuvastatin (TC: atorvastatin vs rosuvastatin = −20.8 ± 3.6 vs. −22.8 ± 5; between groups, *p* 0.133; LDL-C: atorvastatin vs rosuvastatin = −20.3 ± 5.7 vs. 20.8 ± 4.1; between groups, *p* 0.253). The number of patients reaching LDL-C values below the threshold of 70 mg/dL was also similar among patients taking atorvastatin/ezetimibe and those taking rosuvastatin/ezetimibe: 20 (61%) versus 18 (66%), respectively (between groups, *p* 0.074).

## 4. Discussion

Lowering LDL-C below recommended thresholds is a key therapeutic target in the prevention of ASCVD [20]. However, many patients do not reach recommended values of LDL-C despite taking one or more lipid-lowering drugs [12]. In high-risk patients, starting a statin therapy is strongly recommended but, in cases where statins fail to meet LDL-C goals, it is not clear what the next best therapeutic option is to further improve LDL-C control. In this scenario, new non-statin lipid-lowering drugs can represent an alternative strategy for physicians to optimize LDL-C beyond statin titration until the maximal tolerated dose. In the present study, we found that the addition of bempedoic acid to patients already taking a combined therapy of high-intensity statin plus ezetimibe was more effective than doubling the statin dose in lowering LDL-C values. In particular, we observed a 22.9% reduction in LDL-C in the BA group versus a 7.5% reduction in the ST group. We believe that this is an original result for two reasons. Firstly, in the literature we did not find previous studies comparing bempedoic acid versus statin titration; secondly, the addition of bempedoic acid in subjects already taking a statin plus ezetimibe is a therapeutic modality that has so far been little investigated [16]. The LDL-C-lowering effect of bempedoic acid oscillates between 15 and 25% in relation to different clinical scenarios and background therapies. Our result completely agrees with previous studies in which bempedoic acid has been added to ezetimibe or used in monotherapy in patients with hypercholesterolemia and statin intolerance [21,22]. Conversely the LDL-C-lowering effect that we observed in the BA group was greater when compared to the 15–18% reductions described in other studies performed in patients with already established ASCVD, or with multiple CV risk factors, and in which bempedoic acid has been added to statin therapy [23,24]. A possible explanation for the differences between our results and these two latter studies is that the doses of statins they used were higher than in our study. This is because in those studies bempedoic acid or placebo were added to maximally tolerated doses of statins; on the contrary, the statin doses taken by our patients were low and still not optimized. Additionally, other differences should be underlined. In the study of Goldberg et al. [24], for example, only a small proportion of patients were taking ezetimibe in addition to statins and some patients were taking proprotein convertase subtilisin/kexin type 9 inhibitors. Regarding the 7.5% reduction in LDL-C that we observed in the ST group by doubling the dose of statin, this result appears to also be in line with the previous literature: despite the fact that different statins have varying abilities to lower LDL-C, it has been estimated that, on average, doubling the dose of a statin results in an approximate 6% further decrease in LDL-C levels [25]. Interestingly, in this study, the greater reduction in LDL-C observed in the BA group resulted in a significantly higher percentage of patients of this group reaching the therapeutic target at 12 weeks in comparison to the ST group. This result suggests that starting bempedoic acid rather than titrating the statin dose would allow a quicker achievement of optimal values of LDL-C. Further studies are needed to clarify whether the observed differences persist over time and whether the triple strategy is associated with prognostic advantages in comparison to statin titration. Potential prognostic benefits of bempedoic acid in primary prevention have been suggested by a subgroup analysis of CLEAR Outcomes, in which a relative risk reduction of 39% and 27% for cardiovascular and all-cause mortality, respectively, has been demonstrated [17]. Moreover, the prognostic impact of a triple therapy including bempedoic acid, ezetimibe, and maximally tolerated statins has been recently explored. McQueen et al. [26], by using a simulation model, calculated that adding bempedoic acid plus ezetimibe in patients already on maximally tolerated statins and not at their LDL-C goal would result in more major cardiovascular events avoided compared to the addition of ezetimibe alone. Overall, our results suggest that a “triple therapy” strategy, including bempedopic acid, statin, and ezetimibe, could be the most effective for magnifying the LDL-C-lowering effects in high-risk patients. Similar evidence emerges from the study of Ballantyne et al. [27], also conducted in a high-risk population. In that study, the addition of bempedoic acid to statin led to a 17.2% reduction in LDL-C, while the addition of bempedoic acid plus ezetimibe to statin obtained a 34% LDL-C reduction. In our study, the addition of bempedoic acid was well tolerated when added to a background of statin plus ezetimibe. These safety and tolerability findings were consistent with expectations based on previous bempedoic acid clinical trials [28,29]. However, this result should be taken with caution since the follow up period of the present study was very short and data regarding side effects in the literature are not univocal. For example, Ray et al. [23] observed that the incidence of adverse events leading to discontinuation of the drug was higher in the bempedoic acid group than in the placebo group. The results of the present study support the use of a “three drug” approach in primary prevention for two main reasons. Firstly, such approach, by allowing physicians to reach target LDL-C levels in a larger number of high-risk subjects, could have positive repercussions in the prevention of cardiovascular events. Secondly, by avoiding the titration of statins, the three drug approach aims to minimize possible side effects of cholesterol-lowering therapy. Clearly, studies with a longer follow up are mandatory to verify the long-term safety profile of such approach. At the same time, new properly designed trials are needed for exploring the impact of triple therapy on cardiovascular events.

Limitations. The most important limitations of the present study concern its design: the study lacks a control group, and this clearly weakens the robustness of our results. Furthermore, the lack of blindness increases the risk of incurring bias, whereby we cannot rule out that the results we observed could reflect the action of other variables in addition to the effect of the therapeutic intervention tested. Therefore, future double-blinded trials with adequate sample size and including a placebo group are needed in order to confirm and expand our results. In this study, we enrolled patients at high CV risk taking low doses of statins and ezetimibe; therefore, we cannot extend our results to patients at very high-risk taking high doses of statins or to patients taking statins in combination with lipid-lowering drugs other than ezetimibe. Despite this study being open to patients at high CV risk under 50 years, we encountered many difficulties in finding patients under 50 years who met the inclusion criterion of taking the combined treatment of statin plus ezetimibe. Therefore, this age group was underrepresented in this study and our results cannot be generalized to this group.

## 5. Conclusions

Our data suggest that the addition of bempedoic acid to statin–ezetimibe could be a reliable and effective strategy for reaching LDL-C targets in high-risk patients. Further studies are needed to confirm our results and to clarify the clinical implications of this new therapeutic strategy.

## Figures and Tables

**Table 1:** Baseline features of recruited patients according to the two groups’ allocation.

	BA Group (n = 60)	ST Group (n = 60)
Clinical profile		
Age, y	61.7.4 ± 11.2	61.9 ± 7.4
BMI, kg/m^2^	27.7 ± 6.4	28.0 ± 7.2
Males, n (%)	32 (53)	34 (56)
Hypertension, n (%)	39 (65)	41 (68)
Diabetes, n (%)	33 (55)	32 (53)
Active smokers, n (%)	18 (30)	17 (28)
Laboratory		
eGFR, mL/min per 1.73 m^2^	89.3 ± 11.6	79.8 ± 14.1
ALT, U/L	30.7 ± 5.3	31.0 ± 7.9
AST, U/L	30.2 ± 8.2	29.8 ± 8.6
Creatinine, mg/dL	0.91 ± 0.7	0.87 ± 0.3
Uric acid, mg/dL	6.2 ± 1.5	5.8 ± 2.1
CK, U/L	68.3 ± 13.4	75.2 ± 18.1
Glucose, mg/dL	101.2 ± 26.7	97.8 ± 21.84
Treatments		
Atorvastatin, n (%)	33 (55)	31 (54)
Betablockers, n (%)	14 (93.3)	14 (93.3)
ACE-Is /ARBs, n (%)	23 (38)	21 (35)
CCAs, n (%)	16 (27)	19 (32)
Acetylsalicylic acid, n (%)	7 (12)	9 (15)
Clopidogrel, n (%)	3 (5)	4 (6)
Metformin, n (%)	22 (37)	23 (38)
SGLT2-Is, n (%)	18 (30)	17 (28)
Sitagliptin, n (%)	6 (10)	8 (13)

BMI = body mass index; AST = aspartate aminotransferase; ALT = alenineaminotransferase, CK = creatine kinase; ACE-Is = angiotensin-converting enzyme inhibitors; ARBs = angiotensin receptor blockers; CCAs = calcium-channel antagonists; SGLT2-I = sodium-glucose transport protein 2 (SGLT2) inhibitors. At baseline, there were no significant differences between the two groups regarding anthropometric characteristics, prevalence of comorbidities, and pharmacological treatment.

**Table 2:** Changes on lipids in the two study groups (12-weeeks versus baseline).

	BA Group (n = 60)		Δ	ST Group (n = 60)		Δ
	Baseline	12 Weeks		Baseline	12 Weeks	
TC, mg/dL	148.8 ± 44.5	126.9 ± 52.1	−21.9 ± 6.2 *	146.0 ± 48.6	139.5 ± 55.2	−6.5 ± 2.4
LDL-C, mg/dL	89.9 ± 7.9	69.4 ± 6.5 *	−20.5 ± 7.3 *	87.5 ± 8.8	80.8 ± 8.5	−6.7 ± 2.5
HDL-C, mg/dL	37.2 ± 4.6	39.0 ± 3.8	1.8 ± 0.6	36.9 ± 4.2	38.3 ± 5.7	1.4 ± 0.7
TG, mg/dL	108.4 ± 32.4	95.9 ± 39.1	−12.5 ± 3.7	109.2 ± 33.7	99.1 ± 26.4	−10.2 ± 3.1
Non-HDL-C	112.3 ± 26.5	87 ± 25.9	−25.1 ± 7.4 *	109.5 ± 28.3	101.2 ± 21.9	−8.3 ± 2.7

TC = total cholesterol; LDL-C = low-density lipoprotein cholesterol; HDL-C = high-density lipoprotein cholesterol; TG = triglycerides; Non-HDL-C = non-HDL cholesterol. * Between groups, *p* < 0.05. At 12 weeks, observed reductions in total cholesterol, LDL cholesterol and non-HDL cholesterol were significantly greater in the BA (ac bempedoic + statin + ezetimobe) compared to ST (statin titration + ezetimibe) group.

## Data Availability

The data presented in this study are available on request from the corresponding authors.
